# Frosted Branch Angiitis in the Setting of Active COVID-19 Infection and Underlying Mixed Connective Tissue Disease

**DOI:** 10.7759/cureus.36819

**Published:** 2023-03-28

**Authors:** Justin Hanson, Alexander B Dillon, Greg Budoff, Angela J Oh, Kendall Goodyear, Maltish Lorenzo, Steven D Schwartz

**Affiliations:** 1 Department of Ophthalmology, UCLA Jules Stein Eye Institute, Los Angeles, USA; 2 Department of Ophthalmology, East Bay Retina Consultants, Oakland, USA

**Keywords:** viral infection, covid-19, retinal vasculitis, connective tissue disease, frosted branch angiitis

## Abstract

Frosted branch angiitis (FBA) is an uncommon form of retinal vasculitis and is typically associated with vision loss. We report a unique case of FBA that manifested in the setting of an active COVID-19 infection in a patient with Mixed Connective Tissue Disease (MCTD). A 34-year-old female with a history of MCTD, including overlapping findings of dermatomyositis, systemic lupus erythematosus, and rheumatoid arthritis, on immunosuppressive medications, presented for left-sided vision loss. She was also found to have an active COVID-19 infection with symptoms including sore throat and dry cough. The patient's visual acuity was counting fingers in her affected eye with a fundus exam revealing diffuse retinal hemorrhages, retinal whitening, cystoid macular edema, and perivascular sheathing of tertiary arterioles and venules, characteristic of FBA. Labs showed mildly elevated inflammatory markers. She exhibited no other signs or symptoms concerning systemic rheumatologic flare. There was no evidence of COVID-19 on viral PCR testing of intraocular fluid but given her positive nasopharyngeal PCR, COVID-induced retinal vasculitis with FBA remained high on the differential. The patient’s retinal vasculitis later improved with heightened immunosuppressive therapy including high-dose intravenous corticosteroids. Clinicians should be aware of the possibility of COVID-related FBA, particularly in patients with an underlying predisposition to autoimmune inflammation. Our experience with this patient highlights the utility of high-dose systemic immunosuppressive therapy in treating such inflammatory occlusive retinal vasculitis. Further studies are needed to characterize retinal manifestations of COVID-19 in the setting of autoimmune disease.

## Introduction

Frosted branch angiitis (FBA), a severe retinal vasculitis and periphlebitis with a characteristic appearance resembling frosted branches on a tree, was first coined by Ito in 1976 [1]. While the primary form of FBA is idiopathic, secondary forms of the disease may be caused by a viral infection, most notably CMV, or a known autoimmune disease resulting in immune complex deposition giving rise to this phenotype [2,3]. In cases involving infectious etiologies, it is thought FBA may result from direct infection of the retinal vasculature [3].

There have been two reported cases of FBA involving COVID-19 in the literature, the first of which involved a 33-year-old, human immunodeficiency virus-infected patient with coexisting cytomegalovirus (CMV) and COVID-19 infection [4], and the second involving a child with isolated COVID-19 illness and no other relevant medical history [5]. Patients reported to have FBA following viral infection often fall into a bimodal age distribution, where there is a peak in early childhood and another in the second or third decade of life [3]. Herein, we present a rare manifestation of FBA in the setting of active COVID-19 illness in a patient with known and treated chronic connective tissue disease.

## Case presentation

A 34-year-old female with a past medical history of treated MCTD, chronic migraines, treated iron and vitamin D deficiencies, fibromyalgia, and anxiety and depression on SSRIs, presented to the emergency department with acute onset painless loss of vision in her left eye for three days. Her rheumatologic history consisted of Mixed Connective Tissue Disease (MCTD) along with dermatomyositis, systemic lupus erythematosus, and rheumatoid arthritis. The patient’s MCTD was diagnosed in 2014 and managed with hydroxychloroquine (400 mg daily), azathioprine (100 mg daily), and low-dose prednisone (10 mg daily). Her exam was stable over the two years prior. The patient was single at the time of presentation, with history of one unplanned pregnancy and the child is currently one year old. On presentation, she denied symptoms consistent with rheumatological flare (arthralgia, Raynaud’s, hand swelling, heliotrope rash, and proximal muscle weakness). She did, however, complain of nausea and fatigue beginning three days prior to her vision loss in the left eye, followed by a sore throat and dry cough. She tested positive for COVID-19 via polymerase chain reaction (PCR) of the nasopharynx, however aqueous humor PCR for COVID-19 was negative. Anterior chamber tap of the aqueous humor was also negative for CMV, HSV Type 1 and 2, and Varicella Zoster via PCR. HIV and CMV serologies were negative.

**Figure 1:**
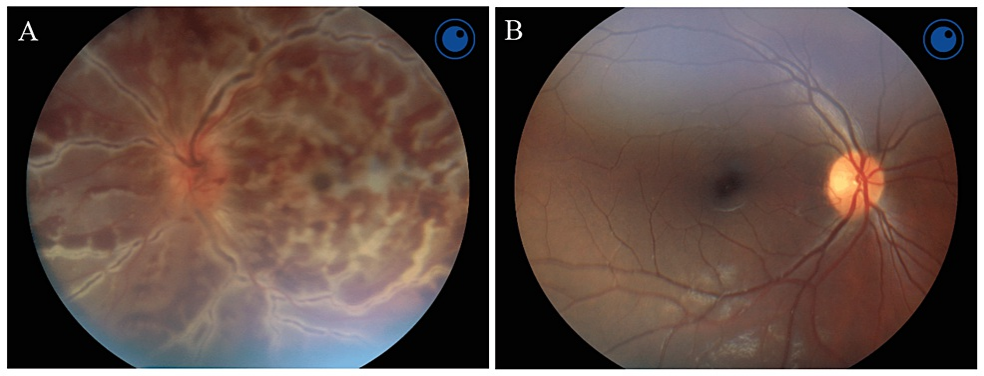
Dilated fundus photography in a patient with frosted branch angiitis three days after symptom onset (A) Left eye photographs show significant optic disc edema with diffuse perivascular sheathing, retinal hemorrhages, and whitening in the macula and periphery. (B) The contralateral right eye appears unremarkable.

Her best corrected visual acuity was 20/30 in the right eye and counting fingers in the left eye. Intraocular pressures were within normal limits. There was a left relative afferent pupillary defect. An anterior chamber exam showed 3+ mixed cells in her left eye only. Dilated fundus examination revealed significant left optic disc edema with diffuse retinal hemorrhages and whitening in the macula and periphery accompanied by marked macular edema (Figure 1A).

Diffuse perivascular sheathing was noted (Figure 1A). Dilated exam of the right eye was unremarkable (Figure 1B). Given her history of autoimmune disease, extensive rheumatology and infectious work-up were completed, and were overall unremarkable save for elevated, non-specific inflammatory markers (C-reactive protein, 2.4 mg/dL [normal <0.3 mg/dL], erythrocyte sedimentation rate, 54 mm/hr [normal <20 mm/hr]). At the time of initial presentation, she was afebrile (36.7°C), blood pressure was 98/77, pulse was 77 bpm, and oxygen saturation 97% on room air. Blood sugar at the time was 101 mg/dL and all electrolytes were within normal limits. Tonometry of the left eye showed 19mmHg. 

While the etiology of her acute retinal vasculitis remained unclear, suspicion remained high for an active role of her acute COVID-19 infection given the temporal association.

**Figure 2:**
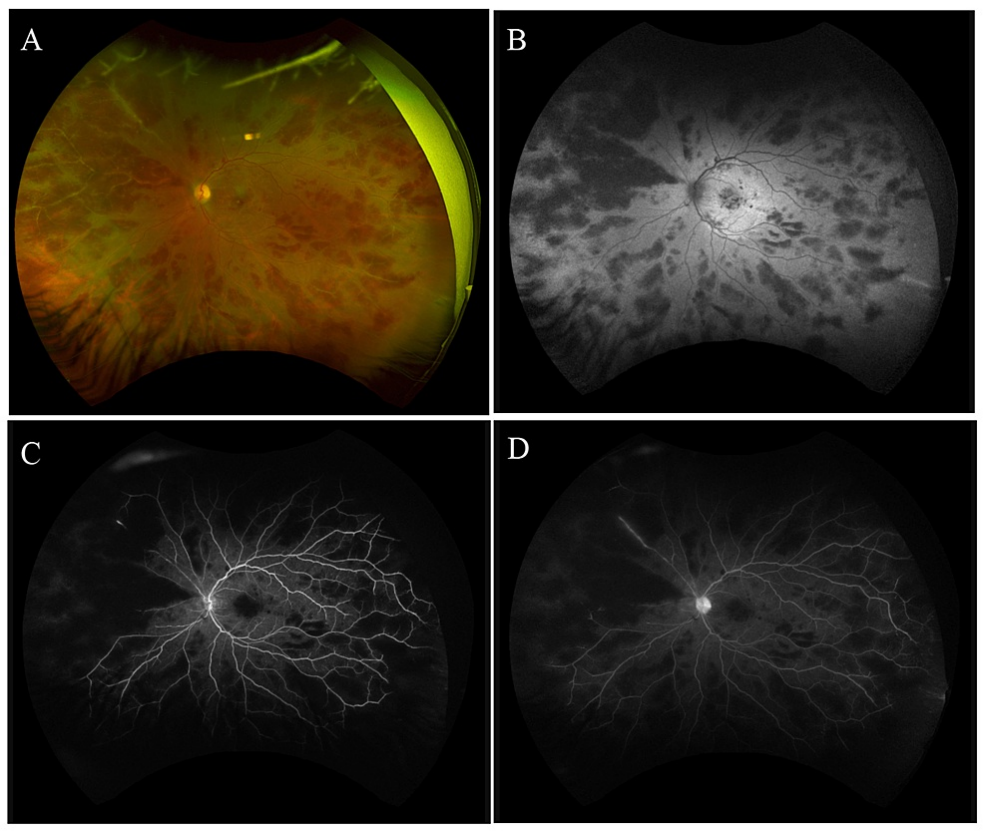
Ultrawide field retinal imaging of the left eye 10 days after initiation of IV pulse-dose steroids in a patient with frosted branch angiitis (A) Marked improvement in optic nerve head edema, retinal vascular tortuosity, diffuse retinal hemorrhages, and sheathing of peripheral retinal vasculature in all quadrants. (B) Fundus autofluorescence shows mixed hyper and hypo-autofluorescence in the macula. (C, D) Fluorescein angiography shows vascular leakage and peripheral ischemia. There is no evidence of neovascularization.

**Figure 3:**
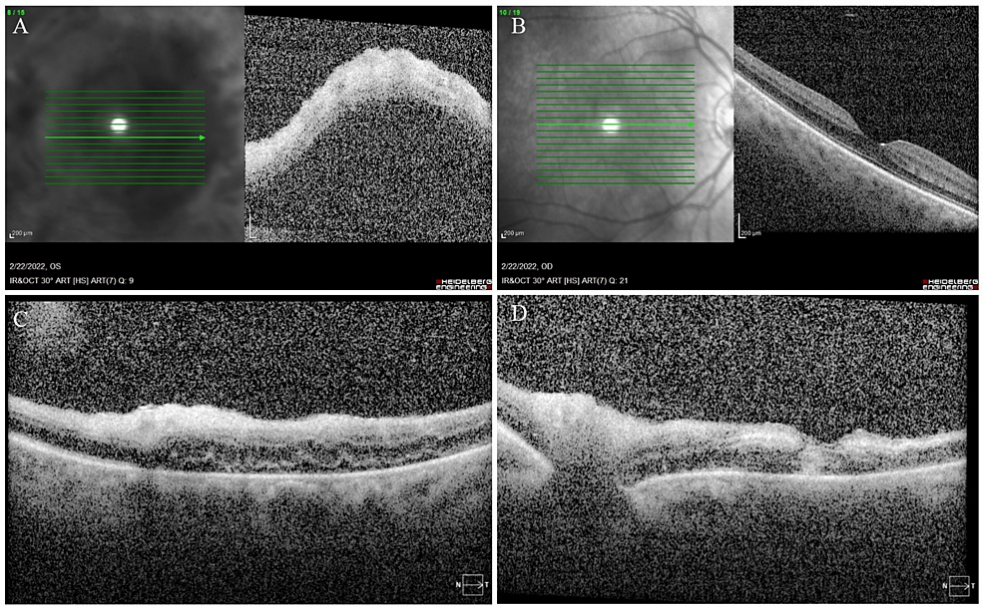
Optical coherence tomography (A) Left eye shows significant edema and subretinal fluid. (B) The fellow right eye is unremarkable. After initiation of intravenous pulse-dose steroids 10 days later, the subretinal fluid has significantly improved but with resultant disorganization of retinal inner layers. (C) A low-lying focal serous retinal detachment is noted in the inferior macula, as well as inner retinal hyperreflectivity. (D) There is mild residual optic nerve head edema and a full-thickness hyperreflective focus on the fovea with resolution of macula edema.

High dose intravenous steroids (methylprednisolone 1g daily for three days), remdesivir, and valacyclovir were initiated, followed by prednisone 70 mg PO daily. No supplemental oxygen was needed during her treatment course. She was also started on topical prednisolone acetate (1 drop to her affected eye QID). Her chronic MCTD regimen was continued (hydroxychloroquine 400 mg daily and azathioprine 100 mg daily). Ten days following initiation of intravenous steroids, her vision remained stable. There was mild subjective improvement, accompanied by a notable reduction in the degree of retinal vasculitis (Figures 2A-2D) and macular edema (Figures 3C, 3D).

Three-week rheumatologic follow-up confirmed the patient showed no other clinical signs of active MCTD. Oral prednisone was decreased to 60 mg daily, azathioprine was discontinued, and mycophenolate (360 mg twice daily for one week) was initiated with plans to titrate to 1080 mg twice daily.

**Figure 4:**
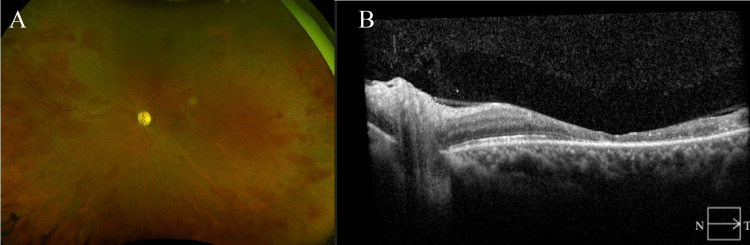
Three-month follow-up showing macular atrophy with resolution of macular edema (A) Ultrawide field retinal imaging and (B) optic coherence tomography imaging of the left eye in a patient with frosted branch angiitis.

At the three-month follow-up, her visual acuity was stable at counting fingers in her affected eye, limited by macular ischemia and atrophy. Her contralateral eye remained uninvolved with 20/20 visual acuity. Dilated fundus exam OS showed significant improvement of retinal hemorrhages after her steroid taper of decreasing from 60 mg to 40 mg over one month and then decreasing by 5 mg every 14 days, to a final dose of prednisone 20 mg PO daily on three-month follow-up. Optical coherence tomography showed resolution of macular edema with significant macular atrophy (Figures 4A, 4B). Fluorescein angiogram showed improving occlusive vasculitis with both macular and peripheral ischemia without neovascularization.

## Discussion

FBA is associated with intraocular infections including CMV, Herpes and Toxoplasmosis, and autoimmune conditions such as systemic lupus erythematosus, Behçet’s disease, and Crohn’s disease. This is thought to be from direct infection of the retinal vessels [2,3] or immune-complex deposition in vessel walls, respectively [2,6]. The characteristic perivascular whitening results from lymphoplasmacytic infiltrates [7]. A primary idiopathic form of the disease also exists that mostly affects younger patients [3].

The FBA phenotype of the present case manifested in the setting of both a history of autoimmune disease and active COVID-19 viral infection. While there was no detectable COVID-19 on aqueous sampling, our patient showed no other active symptoms of the autoimmune disease during her presentation. It is possible that the combination of her viral illness and underlying autoimmune milieu resulted in her FBA. To our knowledge, there is only two other cases of FBA following COVID-19 illness, the first reported in which FBA arose after a co-infection with CMV in a patient with underlying human immunodeficiency virus [4]. Our patient had neither positive serologies for CMV or human immunodeficiency virus. The second reported case involved a pediatric patient with no relevant past medical history and identified SARS-CoV-2 as the sole identifiable cause of the patient’s FBA [5]. The fact that SARS-CoV-2 is the only virus affecting the patient is similar to our case, although the presentation lacks any underlying history of MCTD. While the field is still learning about the ocular sequelae of COVID-19, this case suggests a potential association between COVID-19 and underlying autoimmune disease in the manifestation of this retinal vasculitis.

Viral infections have been shown to play a significant role in autoimmune phenomena in patients with impaired immune regulation, either prompting the onset of autoimmune disease or exacerbating existing disease [8]. For example, there are cases in which viral illness is thought to elicit a new presentation of lupus nephritis [9] or exacerbate multiple sclerosis [10]. In the present case, COVID-19 may have caused an exacerbation of the patient’s MCTD and lead to ocular involvement presenting as FBA. The absence of other active MCTD symptoms in our patient may be attributed to long-term management and control of the overlapping autoimmune diseases with her home immunosuppressive regimen. It is also possible that COVID-19 was the only cause or trigger of FBA, with previous evidence of a pediatric patient with a completely negative laboratory work-up other than positive IgG antibodies to COVID-19 and a recently suspected infection [5].

Previous cases involving secondary FBA suggest systemic steroid therapy can reduce progression of vision loss and in some cases, aid in visual recovery [2,3,6]. Mimura et al describe the case of a 53-year-old Japanese woman with MCTD who developed retinal vasculitis and reduced vision to counting fingers with findings of FBA. Pulse dose steroids and modification of her immunosuppressive regimen was associated with an improvement in her visual acuity to 20/20 in her affected eye [11]. Although our patient’s vasculitis improved on an extensive immunosuppressive regimen, her objective visual acuity did not improve significantly in the setting of macular ischemia.

## Conclusions

Given the complexity of the present case of likely multifactorial secondary FBA, a multidisciplinary approach was necessary to devise a diagnostic and therapeutic plan. This case illustrates the potentially devastating nature of FBA in severe cases despite prompt aggressive treatment. COVID-19 may be a risk factor for development of FBA in patients with underlying autoimmune conditions. Further research is needed to determine the underlying mechanisms through which autoimmune and viral diseases may interact to give rise to vision-threatening FBA.
